# Supplementary material for: Selective interlayer ferromagnetic coupling between the Cu spins in YBa2Cu3O7−x grown on top of La0.7Ca0.3MnO3
Source: Sci Rep. 2015 Nov 17;5:16690. doi: 10.1038/srep16690 (PMC4648077; doi:10.1038/srep16690)
Supplement: Supplementary Information [file srep16690-s1.pdf]

Supplementary Information for "Selective interlayer ferromagnetic coupling between the Cu spins in $YBa_2Cu_3O_{7-x}$ grown on top of $La_{0.7}Ca_{0.3}MnO_3$"

S. W. Huang[1,2,3], L. Andrew Wray[1,4,5], Horng-Tay Jeng[6,7], V. T. Tra[8], J. M. Lee[9], M. C. Langner[2], J. M. Chen[9], S. Roy[1], Y. H. Chu[10], R. W. Schoenlein[2], Y.-D. Chuang[1,*], and J.-Y Lin[8,1,+]

[1]Advanced Light Source, Lawrence Berkeley National Laboratory, Berkeley, CA 94720, USA
[2]Materials Sciences Division, Lawrence Berkeley National Laboratory, Berkeley, CA 94720, USA
[3]MAX IV Laboratory, Lund University, P. O. Box 118, 22100 Lund, Sweden
[4]Department of Physics, New York University, New York, New York 10003, USA
[5]Stanford Institute for Materials and Energy Sciences, SLAC National Accelerator Laboratory, Menlo Park, CA 94025, USA
[6]Department of Physics, National Tsing Hua University, Hsinchu 30013, Taiwan
[7]Institute of Physics, Academia Sinica, Taipei 11529, Taiwan
[8]Institute of Physics, National Chiao Tung University, Hsinchu 30010, Taiwan
[9]National Synchrotron Radiation Research Center, Hsinchu 30076, Taiwan
[10]Department of Materials Science and Engineering, National Chiao Tung University, Hsinchu 30010, Taiwan
[*]ychuang@lbl.gov
[+]ago@cc.nctu.edu.tw

ABSTRACT

I. Structure of YBCO/LCMO bilayers grown by pulse laser deposition (PLD)

The ability to control the growth process down to atomic level is the key to the success of this study. In the PLD system, a 10 Hz 250 mJ pulsed laser beam from KrF (λ = 248 nm) excimer laser was used to evaporate the targets. The (100) oriented $SrTiO_3$ (STO) single crystal was used as the substrate. Before growing the bilayers, the substrate was first treated with HF-NH_4F buffer solution to produce a uniform TiO_2 termination. Depositing $La_{0.7}Ca_{0.3}MnO_3$ on top of this TiO_2 terminated STO substrate produces the MnO_2 termination at the $YBa_2Cu_3O_{7-x}$ - $La_{0.7}Ca_{0.3}MnO_3$ (YBCO-LCMO) interface. To produce the $La_{0.7}Ca_{0.3}O$ termination at the manganite/cuprate interface, a 1.5 unit cell (*u.c.*) $SrRuO_3$ buffer layer was deposited on top of the STO substrate before growing the LCMO layer. The LCMO (YBCO) layer was deposited at 700 ^{o}C (750 ^{o}C), 80 mTorr (150 mTorr) oxygen pressures. After growth, the bilayer samples were further annealed in a 550 ^{o}C, 700 Torr oxygen atmosphere for one hour followed by a slow cool down to the room temperature to achieve the full oxygenation for the YBCO layer. We used the *in-situ* reflection high-energy electron diffraction (RHEED) to monitor the growth process. Clear RHEED oscillations confirmed the layer-by-layer growth mode (for examples, see Fig. S1). To determine the quality of YBCO-LCMO interface, high angle annular dark-field scanning transmission electron microscopy (HDDAF-STEM) was used. The HDDAF-STEM images confirmed the structures illustrated in Fig. 1(b) in the manuscript.[1]

II. Transport properties of YBCO/LCMO bilayers

The resistivity of bilayer samples was measured using the standard four-probe method. The results are shown in Fig. S2(a) as well as in Fig. 3(d) in the manuscript. The superconducting transition temperatures are determined to be $\sim$ 70 and 55 K for MnO_2 and $La_{0.7}Ca_{0.3}O$ terminated bilayers respectively. The Curie temperature, which is the onset temperature of ferromagnetism in the LCMO layer, is estimated by intersecting the linear extrapolation of the high temperature leading edge of $dM(T)/dT$ curve to zero. The extrapolation is shown as the dashed line in Fig. S2(b) and the Curie temperature is estimated to be 190 K, with an uncertainly on the order of 10 K.

In Fig. 3(b) in the manuscript, the onset of RSXS intensity (red markers) is slightly higher than the Curie temperature (open

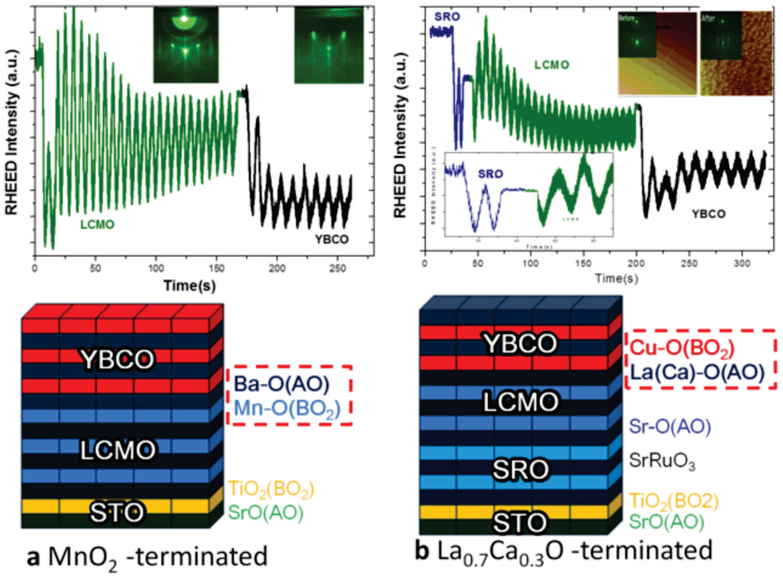

Figure 1. (color online) Examples of the growth of (a) the STO/LCMO$_{10nm}$/YBCO$_d$ structure with MnO_2-terminated interface and (b) the STO/SRO$_{1nm}$/LCMO$_{10nm}$/YBCO$_d$ structure with $La_{0.7}Ca_{0.3}O$ terminated interface. Top panels show the layer-by-layer growth mode monitored by the RHEED.

arrow) and this can be understood as the following. For magnetization, the recorded signal is the sum of magnetic moments from different ferromagnetic domains. When these magnetic domains are not properly aligned, say pointing along the easy axes (Mn-O bond direction) but antiparallel to each other, cancellation can lead to a smaller reading in $M(T)$. On the other hand, the RSXS intensity measures primarily the sum of the square of these moments. Thus even in this scenario, as long as the c-axis ferromagnetic coupling is established, the RSXS intensity from these anti-aligned domains will add up. Thus it is plausible that in the temperature regime between 200 K and 150 K, the discrepancy between $M(T)$ and RSXS data is caused by the intricate re-alignment of microscopic ferromagnetic domains. Moreover, a short range ordering preceding the establishment of bulk ferromagnetism would also lead to a higher onset temperature for the RSXS intensity than the Curie temperature by a similar mechanism as mentioned above.

III. Two-peak structures in the resonance profile of YBCO (001) Bragg peak

In Fig. 2 of the manuscript, we show the resonance profiles (thick solid lines) of YBCO (001) Bragg peak from MnO_2 and $La_{0.7}Ca_{0.3}O$ terminated bilayers. Unlike pure YBCO film which shows just one feature at Cu L_3 and L_2 edges, bilayer samples exhibit two peaks at these edges (the additional peak at L_3 edge is labelled B).

The two-peak structure is intrinsic to the bilayers. Although one might speculate that it could come from two types of Cu^{2+} with different binding energies, this scenario can be ruled out as the similarity between the XAS spectra from these three samples does not support two distinct Cu^{2+} states with such large energy difference ($\sim$ 2.5 eV apart). Furthermore, we also try to simulate the RSXS spectra of bilayers by using the ones from pure YBCO film with a 2.5 eV relative energy shift. Although the simulated spectrum (black curve, Fig. S3) seems to capture the gross spectral lineshape, the differences can still be clearly seen at selected photon energies (see arrows in Fig. S3).

The self-absorption effect, where x-rays emitted from deep inside the sample are re-absorbed when they come out of the sample, tends to suppress the high intensity features in XAS spectra recorded in the fluorescence yield mode. This effect becomes appreciable around the elemental absorption edges at which the x-ray penetration depth is significant reduced. Self-absorption correction is often performed when the x-ray penetration depth is comparable to or shorter than the thickness of sample. In our case where the thickness of YBCO film is around 15 nm (at 35° grazing incidence angle, the effective thickness is around 26 nm) and the minimum x-ray penetration depth at Cu L_3 edge is around 140 nm (attenuation length determined from the CXRO website; http://henke.lbl.gov/optical_constants/), the self-absorption effect is not expected to significantly alter the intensity ratio between features A and B. Thus it cannot be used to account for the observed two-peak structure in RSXS data.

The intensity of XAS is proportional to the imaginary part of atomic scattering form factor $f^{''}(E)$, whereas the RSXS intensity is related to its square $(f^{'}(E))^2+(f^{''}(E))^2$ modulated by a phase factor from the spatial arrangement of these scatterers

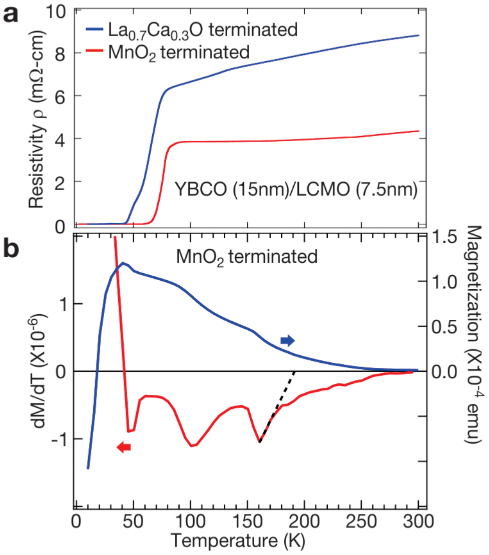

Figure 2. (color online) (a) The in-plane resistivity of MnO_2 (red curve) and $La_{0.7}Ca_{0.3}O$ (blue curve) terminated bilayers showing the respective superconducting transition temperatures $T_c \sim 70$ K and 55 K determined by the mid-point of the transition. (b) Magnetization (blue curve, right axis) and its first derivative (red curve, left axis) from MnO_2 terminated bilayer. The dashed line indicates the linear extrapolation of the leading edge of the red curve.

(see description in the manuscript). $f^{'}(E)$ and $f^{''}(E)$ are related to each other through Kramers-Kronig relations. We notice that the intensity of YBCO (001) Bragg peak is much weaker than other $(00L)$ Bragg peaks and this is due to an effective destructive interference between Cu charge scatterings from the CuO chain and two CuO_2 planes within the unit cell. Such interference can be disrupted by a slight shift in the resonance energies or variations in the spatial arrangement of the scatterers. The former one will affect the energy denominator in $f^{'}(E)$ and $f^{''}(E)$, whereas the later one will affect the phase factor. Simulating the RSXS lineshape will require the full knowledge of the spatial arrangement of Cu charges within the unit cell and their energetic upon heterogeneity, but to lowest order, these two factors can explain the relative intensity change between feature A and B in Fig. 2 in the manuscript. Irrespective to which origin, the distinct RSXS resonance profiles seen in the bilayer samples implies that the local energetic of electronic states is altered upon heterostructuring.

IV. Azimuthal angle dependence of the RSXS intensity

Unlike the magnetization, RSXS has the unique elemental, chemical and bonding specificity to differentiate the origins of the magnetic moments. Complementary to the x-ray magnetic circular dichroism (XMCD), RSXS intensity dependence on the tensorial nature of scattering channels can be helpful in identifying the magnetic couplings between the CuO_2 planes when the ordering vector overlaps with the structural Bragg peak whose intensity is dominated by the charge scattering.

In the current study, the scattering plane is horizontal and the incident photon polarization is in this scattering plane (π-polarization). We have used the single channel detector (photodiode) without polarization analyzer to record the scattering signal. The recorded signal will contain both σ- and π-polarization components. Although this may complicate the analysis of spin states, we will show that it still can offer some useful insight.

We follow the formalism outlined in Hill & McMorrow[2] and Lovesey & Collins.[3] Since the incident photon energy is tuned close to Cu^{2+} L_3 absorption edge, we only consider the dipole ($E1$) transition and neglect the much weaker quadrupole ($E2$) transition. Equation (15) in ref[2] is reproduced here:

$$\begin{pmatrix} f_{\sigma_i\to\sigma_o} & f_{\pi_i\to\sigma_o} \\ f_{\sigma_i\to\pi_o} & f_{\pi_i\to\pi_o} \end{pmatrix} = F^{(0)} \begin{pmatrix} 1 & 0 \\ 0 & cos(2\theta) \end{pmatrix} + iF^{(1)} \begin{pmatrix} 0 & z_1 cos(\theta) + z_3 sin(\theta) \\ z_3 sin(\theta) - z_1 cos(\theta) & -z_2 sin(2\theta) \end{pmatrix} + F^{(2)} \begin{pmatrix} z_2^2 & -z_2(z_1 sin(\theta) - z_3 cos(\theta)) \\ z_2(z_1 sin(\theta) + z_3 cos(\theta)) & -cos^2(\theta)(z_1^2 tan^2(\theta) + z_3^2) \end{pmatrix} \quad (1)$$

with $F^{(0)}$,$F^{(1)}$,and $F^{(2)}$ defined in ref.[2] We need to include these three terms because the Kronecker δ that conserves the wave

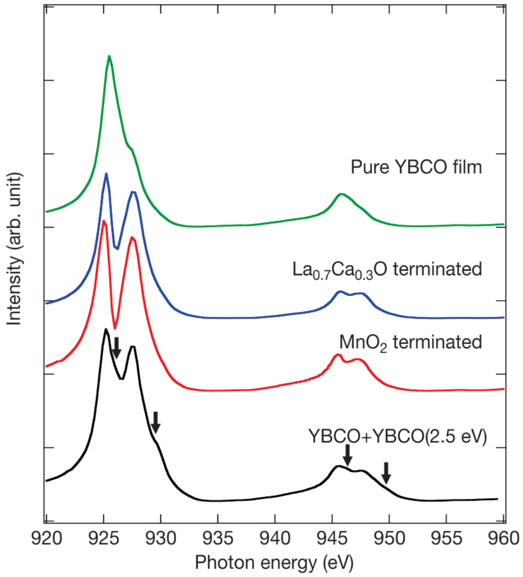

Figure 3. (color online) Resonance profiles of YBCO (001) Bragg peak and simulated spectrum. The black curve is produced by the sum of two spectra from pure YBCO (green curve) with a 2.5 eV relative energy shift. The discrepancies between the simulated spectrum and the ones from bilayers (red and blue curves for MnO_2 and $La_{0.7}Ca_{0.3}O$ terminated bilayers) are highlighted by arrows.

vectors becomes 1 in this case. We only need to consider the second column in the matrix because these elements are relevant to the signal in the $\pi_i \rightarrow \sigma_o$ and $\pi_i \rightarrow \pi_o$ channels (here, π_i and σ_o refer to the incident π and scattered σ polarizations). θ is the YBCO (001) Bragg angle and is $\sim 34.79^o$ in the current study. z_i are the components of spin unit vector projected onto three crystalline axes. They are:

$$\begin{aligned} z_1 &= sin(\alpha)cos(\phi) \\ z_2 &= sin(\alpha)sin(\phi) \\ z_3 &= cos(\alpha) \end{aligned} \tag{2}$$

Here α is the angle between the unit vector and c-axis, and ϕ is the sample azimuthal angle. Firstly, it is clear that if the moment is completely along the c-axis ($\alpha = 0^o$), there will be no azimuthal angle dependence in the RSXS intensity. To simplify the discussion, we consider the extreme case where $\alpha = 90^o$. Putting these terms together, we have:

$$f \sim \begin{pmatrix} f_{\sigma_i \rightarrow \sigma_o} & -i(z_1 cos(\theta))F^{(1)} - z_2(z_1 sin(\theta))F^{(2)} \\ f_{\sigma_i \rightarrow \pi_o} & F^{(0)}cos(2\theta) + iz_2 sin(2\theta)F^{(1)} - z_1^2(sin^2(\theta))F^{(2)} \end{pmatrix} \tag{3}$$

The RSXS intensity is proportional to sum (over the superlattice) of the square of f

$$\begin{aligned} |f|^2 = |F^{(0)}|^2\{ & cos^2(2\theta) \\ & - \frac{2\Im(F^{(0)*}F^{(1)})}{|F^{(0)}|^2} cos(2\theta)sin(2\theta)sin(\phi) \\ & - \frac{2\Re(F^{(0)*}F^{(2)})}{|F^{(0)}|^2} cos(2\theta)sin^2(\theta)cos^2(\phi) \\ & + \frac{2\Im(F^{(2)*}F^{(1)})}{|F^{(0)}|^2}(2sin^2(\theta)-1)sin(\theta)cos(\theta)cos^2(\phi)sin(\phi) \\ & + \frac{|F^{(1)}|^2}{|F^{(0)}|^2}(cos^2(\theta)cos^2(\phi) + sin^2(2\theta)sin^2(\phi)) \\ & + \frac{|F^{(2)}|^2}{|F^{(0)}|^2}(sin^4(\theta)cos^4(\phi) + sin^2(\theta)sin^2(\phi)cos^2(\phi))\} \end{aligned} \tag{4}$$

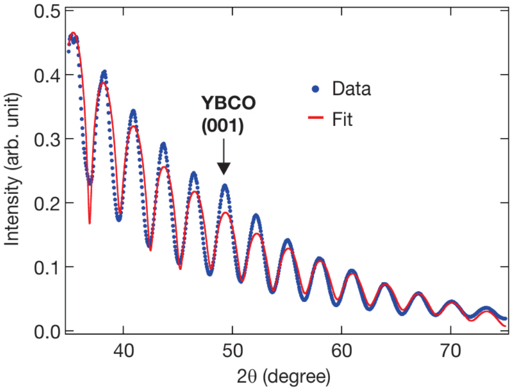

Figure 4. (color online) Synchrotron reflectivity measurement. The agreement between data (blue markers) and fit (red line) implies that the choice of 15 nm YBCO and 7.5 nm LCMO layer thickness with 0.6 nm roughness in fitting is reasonable. The discrepancy around 50° is due to the presence of YBCO (001) Bragg peak.

Usually, $F^{(0)}$ is much larger than $F^{(2)}$ and $F^{(1)}$ so that the ferromagnetic signal would be very weak compared to the charge signal in the Bragg peak. However, the destructive interference between charge scatterings leads to a much weaker YBCO (001) Bragg peak (see previous discussion). This makes the ratio $\Sigma_{superlattice}[F^{(1,2)}/F^{(0)}]$ not so negligible. But even so, we do not expect the ratio can be on the order of 1. Thus we argue that the high order terms (last three terms in the equation) can be dropped out in the following discussion. By doing so, the scattering intensity will have the azimuthal angle dependence of $a+b*sin(\phi)+c*cos^2(\phi)$ where the coefficients b and c are related to $\frac{F^{(1)}}{F^{(0)}}$ and $\frac{F^{(2)}}{F^{(0)}}$.

From Fig. 3(f) of the manuscript, the Bragg peak intensity changes from ~ 1.5 at 0° to ~ 1.1 at 90° above the charge background of ~ 1.0. Having the spin moment along the c-axis would increase the constant base line and reduce the $[F^{(1,2)}/F^{(0)}]$ ratio. The strong sinusoidal oscillation implies that the in-plane spin component is larger than the out-of-plane component. It also suggests that $F^{(1)}$ is much smaller than $F^{(2)}$, as expected from the extremely weak Cu XMCD versus XLD signal.

Although YBCO has CuO chains that naturally break the four-fold symmetry, the bilayer samples under study are twinned. It is possible that the twinned domains with two distinct CuO chain orientations have unequal volume fractions that give the observed two-fold symmetry, we also notice that the 80 K measurement temperature is below the structural distortion of underlying STO substrate around 105 K.[4,5] This distortion naturally breaks the four-fold symmetry and further aligns the ferromagnetism in LCMO layer as shown in the magnetization.

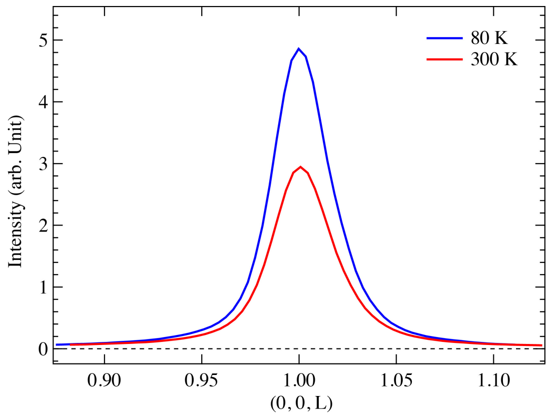

Figure 5. (color online) q-scans at 80 K (blue) and 300 K (red) from the MnO_2 terminated bilayer at 925.25 eV. The photodiode intensity was normalized by the incident photon flux only (photocurrent from upstream Au mesh), and no background subtraction was applied.

V. Determining the layer thickness using synchrotron reflectivity

Synchrotron reflectivity is used to determine the YBCO and LCMO layer thickness in the heterostructures. The measurement temperature was set to 80 K and the incident photon energy was tuned to 1240 eV, well above the Mn and Cu resonances. The blue markers in Fig. S4 are the data while the red curve is the fitting with YBCO / LCMO layer thickness of 15 nm / 7.5 nm respectively with roughness around 0.6 nm. The agreement between data and fit justifies the fitting parameters of layer thickness and roughness. Note that the discrepancy around $2\theta \sim 50^{o}$ is caused by the YBCO (001) Bragg peak.

The Kiessig fringes that overwhelm the YBCO (001) Bragg peak become negligible when the incident photon energy is tuned to Cu L_3 resonance. As can be seen in Fig. S5, the spectra recorded in q-scan can be nicely fitted by a Lorentzian function on top of a monotonic background.

References

1. Tra, V.T. *et al.*, Termination control of charge transfer in $YBa_2Cu_3O_{7-x}/La_{0.7}Ca_{0.3}MnO_3$ heterostructures, Submitted to Adv. Mater.
2. Hill, J. & McMorrow, D. F., X-ray resonant exchange scattering: polarization dependence and correlation functions, Acta Cryst. A **52**, 236-244 (1996).
3. Lovesey, S. W. and Collins, S. P., X-ray scattering and absorption by magnetic materials, Oxford series on synchrotron radiation, Clarendon Press, Oxford 1996.
4. Bell, R. O. and Ruppercht, G., Elastic constants of Strontium Titanate, Phys. Rev. **129**, 90-94 (1963).
5. Loetzsch, R. *et al.*, The cubic to tetragonal phase transition in $SrTiO_3$ single crystals near its surface under internal and external strains, Appl. Phys. Lett. **96**, 071901 (2010).
